# Efficacy and clinical predictors of response to rTMS treatment in pharmacoresistant obsessive-compulsive disorder (OCD): a retrospective study

**DOI:** 10.1186/s12888-020-02769-9

**Published:** 2020-07-16

**Authors:** Reza Rostami, Reza Kazemi, Arezoo Jabbari, Azam Sadat Madani, Hosseinreza Rostami, Mohammad Amin Taherpour, Parviz Molavi, Nematollah Jaafari, Min-Fang Kuo, Carmelo M. Vicario, Michael A. Nitsche, Mohammad Ali Salehinejad

**Affiliations:** 1 Department of Psychology, University of Tehran, Tehran, Iran; 2 Atieh Clinical Neuroscience Centre, Tehran, Iran; 3 Department of Psychology, University of Shahed, Tehran, Iran; 4 Department of Psychiatry, Fatemi Hospital, School of Medicine, Ardabil University of Medical Sciences, Ardabil, Iran; 5 Unité de Recherche Clinique Intersectorielle en Psychiatrie Pierre Deniker, Centre Hospitalier Henri Laborit, 86021 Poitiers, France; 6 Univ. Poitiers & CHU Poitiers, INSERM U1084, Laboratoire Expérimental et Clinique en Neurosciences, 86021 Poitiers, France; 7 Department of Psychology and Neurosciences, Leibniz Research Centre for Working Environment and Human Factors, Dortmund, Germany; 8 University of Messina, Department of Cognitive Science, Messina, Italy; 9 Department of Neurology, University Medical Hospital Bergmannsheil, Bochum, Germany; 10 Ruhr-University Bochum, International Graduate School of Neuroscience, Bochum, Germany

**Keywords:** Obsessive-compulsive disorder (OCD), Repetitive transcranial magnetic stimulation (rTMS), Clinical predictors, Demographic predictors, Yale-Brown obsessive-compulsive scale (Y-BOCS)

## Abstract

**Background:**

Application of repetitive transcranial magnetic stimulation (rTMS) for treating obsessive-compulsive disorder (OCD) has been promising and approved by the Food and Drug Administration in 2018, but effects differ between patients. Knowledge about clinical predictors of rTMS response may help to increase clinical efficacy but is not available so far.

**Methods:**

In a retrospective study, we investigated the efficacy of rTMS over the dorsolateral prefrontal cortex (DLPFC) or supplementary motor area (SMA) in 65 pharmaco-resistant OCD outpatients recruited for rTMS treatment from July 2015 to May 2017. Patients received either SMA rTMS (*n* = 38) or bilateral DLPFC rTMS (*n* = 27) in case of reporting higher affective and depressive symptoms in addition to the primary OCD symptoms. OCD symptoms and depression/anxiety states were measured at baseline (before the 1st session) and after the 20th session of rTMS. Additionally, we performed a binary logistic regression analysis on the demographic and clinical variables based on the Yale-Brown Obsessive-Compulsive Scale (Y-BOCS) 3-factor and 2-factor models and individual items to investigate potential predictors of rTMS response.

**Results:**

Patients’ scores in Y-BOCS and Beck anxiety/depression inventories were significantly decreased following rTMS treatment. 46.2% of all patients responded to rTMS, based on the criterion of at least a 30% reduction in Y-BOCS scores. There was no significant difference between response rates of patients in DLPFC and SMA groups. No significant demographic predictors of rTMS efficacy were identified. The factors “obsession severity”, “resistance” and “disturbance” and the “interference due to obsessions” and “resistance against compulsions” items of the Y-BOCS significantly predicted response to rTMS.

**Conclusions:**

In patients with less intrusive/interfering thoughts, and low scores in the “obsession severity”, “disturbance”, and “resistance” factors, rTMS might have superior effects. Identifying clinical and non-clinical predictors of response is relevant to personalize and adapt rTMS protocols in pharmaco-resistant OCD patients. Interpretation of rTMS efficacy should be done with caution due to the lack of a sham intervention condition.

## Background

With a lifetime prevalence of 1–3% [1], obsessive-compulsive disorder (OCD) is a frequent and disabling psychiatric disorder. It is characterized by intrusive, anxiety-provoking, interfering thoughts (obsessions), and associated repetitive behaviors (compulsions) [2]. OCD, which is frequently undertreated [3], is remarkably heterogeneous in etiology, symptoms, subtype and treatment response [4, 5]. As a result, approximately 40–60% of OCD patients remain treatment-refractory to current first-line therapies [6–8], possibly due to the sub-optimal and non-adapted treatment in non-responders. Accordingly, the development of novel therapeutic strategies based on an improved understanding of OCD pathophysiology is relevant [3, 9].

Previous studies in humans and animal models suggest that functional abnormalities of the cortico-striato-thalamo-cortical circuits and supplementary motor area (SMA) might be central pathophysiological components of OCD [10–13]. The dorsolateral and dorsomedial prefrontal cortex (DLPFC, DMPFC), orbitofrontal cortex (OFC) and anterior cingulate cortex are also proposed to be involved. Involvement of such a diverse regions suggests that the pathophysiology of OCD is heterogeneous which might be an important source of variability in the efficacy of conventional OCD treatments. Neuromodulatory treatments involving brain stimulation can modulate respective target regions in OCD and other disorders with abnormalities related to these regions such as drug addiction, depression, schizophrenia, borderline personality disorder, and ADHD [14–19]. They have also potential for individualization via the informed choice of respective targets. Repetitive transcranial magnetic stimulation (rTMS) is a non-invasive brain stimulation technique proposed as a promising method for treating OCD via altering excitability of DLPFC and SMA [20–22]. TMS alters neural activity and excitability of targeted brain regions [23, 24]. Repetitive application of TMS (i.e., rTMS) can induce neuroplastic after-effects in target areas, and depending on the specific stimulation protocol, the effects can be inhibitory or excitatory [25, 26]. These neuroplastic effects are the main rationale behind the clinical therapeutic effects of rTMS [26, 27].

Previous rTMS studies reported an average response rate of 35% in OCD patients, defined as a minimum of 25–40% reduction in post-treatment symptoms [20]. Higher response rates and augmented efficacy were recently reported in patients with a more homogenous pathological profile, including common pathophysiological deficits [9, 28, 29]. This implies the potential relevance of predictors of effective rTMS treatment in OCD, and accordingly the need for personalizing rTMS treatment based on the pathophysiological and clinical profiles of the patients [28]. In this line, recent reviews of rTMS studies show that brain state-dependent modulatory effects of rTMS are an additional parameter that may potentially affect rTMS effects [30], and taking this factor into account might improve treatment outcomes in patients who usually develop treatment-resistant illness subtypes. Moreover, different cortical regions have been targeted in previous studies with mixed results [31–35], leaving the question of which cortical regions to stimulate unanswered.

While specific stimulation parameters and neurobiological predictors of response to rTMS treatment have been investigated in OCD [9, 36], the importance of clinical and demographic factors has not been systematically explored yet. These factors, especially clinical predictors, play a potentially key role in accurately selecting patients for rTMS treatment. Findings from rTMS studies in other neuropsychiatric disorders suggest that specific symptoms, subtypes or psychological states can distinguish between responders and non-responders to rTMS. We recently investigated clinical and demographic predictors of rTMS response in depressive disorders and found that cognitive-affective symptoms, as compared to somatic symptoms, significantly predict response to rTMS [37]. Another study found that nonresponders to rTMS treatment for depression had markedly higher baseline anhedonia symptoms [38]. Although recent studies tried to predict response to rTMS treatment in OCD based on electrophysiological measures [33], clinical predictors of response so far have not been explored.

In the present study, we aimed to investigate the efficacy of rTMS over two potentially involved cortical regions (i.e., SMA and DLPFC) for reducing OCD symptoms. More importantly, we aimed to identify potential clinical and demographic predictors, that could distinguish between rTMS responders and nonresponders in OCD. Based on previous findings about the efficacy of rTMS in OCD patients [20], we expected to observe a response rate of 35–55%, based on a 30% reduction of the Yale-Brown Obsessive-Compulsive Scale (Y-BOCS) baseline score (responder criterion). With regard to clinical predictors of rTMS response, we aimed to identify predictability of the rTMS effects in OCD by clinical variables (based on Y-BOCS factors and items) and demographic characteristics.

## Methods

### Study design

We retrospectively analyzed a dataset of pharmaco-resistant OCD outpatients who received an rTMS treatment between July 2015 and May 2017. Patients were referred to the Atieh Clinical Neuroscience Center in Tehran, Iran, to receive rTMS. The center admits patients with psychiatric disorders (e.g. depression, OCD), neurological disorders (e.g. stroke, dementia), and pediatric neurodevelopmental disorders (e.g. attention-deficit hyperactivity disorder, autism, learning disabilities).

### Participants

Sixty-five pharmaco-resistant OCD outpatients (Mean age = 32.25, *SD* = 10.23, 35 females) were included in this report. 69 patients were initially included, but four patients did not either finish the treatment course without any reported reason or meet the minimum symptom severity to be included. Of 65 patients 38 patients underwent SMA rTMS protocol and 27 patients received DLPFC rTMS protocol (see Table 2 for more details). A priori sample size calculation showed that based on a medium effect size (f = 0.5) which is suggested for NIBS studies [39], a critical *p*-value of 0.05, and a critical power level of 0.95, the required sample size is 42. The OCD diagnosis was based on the Structural Clinical Interview by a licensed psychiatrist according to the DSM 5 diagnostic criteria, confirmed by patient scores on the Y-BOCS [40]. The inclusion criteria were: (1) 18–65 years old, (2) current OCD diagnosis based on DSM-5 (3) moderate to severe OCD score on the Y-BOCS (scores 16 and higher) (4) response failure to previous or current use of medication/psychotherapy (response failure was defined as scores > 16 at Y-BOCS despite at least two SSRI trials of adequate dosage and duration) and (5) stable medication regimen 8-10 weeks before the intervention and unchanged during the treatment (4–6 weeks) [9]. Exclusion criteria included previous treatment with electroconvulsive therapy, and presence or history of psychosis, substance abuse, suicide attempt and/or active suicide ideation, neurological disorder, epilepsy, seizures, and head injury or loss of consciousness. According to the safety criteria for rTMS [41], patients with implanted devices, metal bodies, cardiac arrhythmia, unstable medical conditions, or pregnancy, were also excluded. 51 patients were taking selective serotonin reuptake inhibitors (SSRIs) during rTMS treatment, and the remaining patients had a history of SSRI medication use. Most of the patients had no history of psychotherapy. All patients provided written informed consent to treatment. Demographic and clinical characteristics of patients are summarized in Tables 1 and 2.

**Table 1:** Demographic information of participants

Variable	Category	*n*	Response (≥30%)		***p***-value
			Yes	No	
**Sample size (*n*)**		65	30	35	
**Comorbidity (*yes*)**		34	12	22	0.06
**On medication (yes)**		51	24	27	0.55
**Protocol**	SMA rTMS	38	16	22	0.30
	DLPFC rTMS	27	14	13	
**Age**	Mean (SD)	32.25 (10.23)	32.67 (9.44)	31.89 (10.99)	0.76
**Gender**	Male	30	14	16	0.56
	Female	35	16	19	
**Education**	Diploma or lower	24	10	14	0.65
	Associate degree	7	3	4	
	Bachelor degree	22	11	11	
	Masters degree	6	2	4	
	Not reported	6	4	2	
**Marital status**	Single	26	10	16	0.71
	Married	33	17	16	
	Divorced or separated	6	3	3	

*Note*: *SMA* Supplementary motor area, *rTMS* repetitive transcranial magnetic stimulation, *DLPFC* dorsolateral prefrontal cortex, *SD* Standard Deviation. Between group (responders vs non-responders) differences in demographic variables were explored using chi-square analysis or Fisher’s exact test for categorical variables and t-tests for continuous variables. Response rate defined as at least 30% reduction of the Yale-Brown Obsessive-Compulsive Scale post-intervention.

**Table 2:** Group (SMA vs DLPFC rTMS) charactristics

Variable	Category	*n*	rTMS protocol		*p*-value	*95%CI*			
			SMA rTMS	DLPFC rTMS		lower		upper	
						SMA	DLPFC	SMA	DLPFC
**Sample size (n)**		65	38	27					
**Comorbidity (n)**		34	20	14	0.09				
**On medication (n)**		51	21	30	0.77				
**rTMS reponse**	Yes	30	21	9	0.12				
	No	35	17	18					
**Age**	Mean (SD)	32.25 (10.23)	31.58 (10.64)	33.19 (9.75)	0.54	27.03	39.49	33.00	44.38
**Gender**	Male	30	17	13	0.80				
	Female	35	21	14					
**Education**	Diploma or lower	24	14	10	0.89				
	Associate degree	7	4	3					
	Bachelor degree	22	13	9					
	Masters degree	6	3	3					
	Not reported	6	4	2					
**Marital status**	Single	26	15	11	0.94				
	Married	33	20	13					
	Divorced or separated	6	3	3					
**BL Y-BOCS**	Mean (SD)	22.75 (6.66)	23.52 (7.37)	21.66 (5.44)	0.27	18.74	26.52	17.07	23.88
**BL BAI**	Mean (SD)	25.90 (11.01)	24.71 (10.15)	27.59 (12.10)	0.32	20.19	30.74	20.47	33.83
**BL BDI-II**	Mean (SD)	22.64 (8.95)	21.84 (9.86)	23.77 (7.54)	0.39	18.36	27.07	21.70	29.98

*Note*: *SMA* Supplementary motor area, *rTMS* repetitive transcranial magnetic stimulation, *DLPFC* dorsolateral prefrontal cortex, *SD* Standard Deviation, *Y-BOCS* Yale-Brown Obsessive-Compulsive Scale, *BAI* Beck Anxiety Inventory, *BDI-II* Beck Depression Inventory; Group differences based on the applied protocol (SMA vs DLPFC rTMS) in demographic variables were explored using chi-square analysis or Fisher’s exact test for categorical variables and t-tests for continuous variables

### rTMS treatment parameters

RTMS was administrated with a Neuro MS rTMS device (Neurosoft, Russia) using a 70-mm figure-of-8-shaped coil (air film coil). Active motor threshold (AMT) was defined as the minimum stimulus intensity that produced a liminal motor evoked response during active contraction of the abductor policies brevis muscle (APB) (at about 20% maximum contraction) [42]. Motor threshold determination was based on visual inspection of the respective finger movement. Patients received either SMA rTMS, or bilateral DLPFC rTMS. For SMA rTMS, the coil was positioned over the SMA, which was localized via the 10–20 EEG system, and defined as 15% of the distance between nasion and inion anterior to the vertex in the sagittal plane [43]. In the SMA-rTMS protocol, TMS was delivered at 120% of AMT. Stimulation frequency was 1 Hz, which was applied for 30 min, resulting in a total of 1800 pulses per session. Stimulation was performed once a day, 3 days per week for 7 weeks, resulting in 20 sessions (36,000 pulses over 20 sessions). For DLPFC rTMS, all patients received bilateral stimulation, based on results of previous studies that showed mixed effects of unilateral rTMS [35]. For DLPFC rTMS, the position of the coil was 5 cm anterior along a parasagittal line from the site of optimum APB stimulation [44]. Stimulation was delivered over the right and left DLPFC respectively. First, 15 min of 1 Hz stimulation (inhibitory) at 120% AMT, resulting in a total of 900 pulses per session, was applied over the right DLPFC, resulting in a total of 18,000 pulses over 20 sessions. The left DLPFC was stimulated immediately afterwards by applying 10 Hz stimulation (excitatory) at 120% AMT via 60 stimulation trains of a duration of 5 s each, with 10 s inter-train intervals, resulting in a total of 3000 pulses per session in 15 min (60,000 pulses over 20 sessions).

### Clinical procedure

**Fig. 1:**
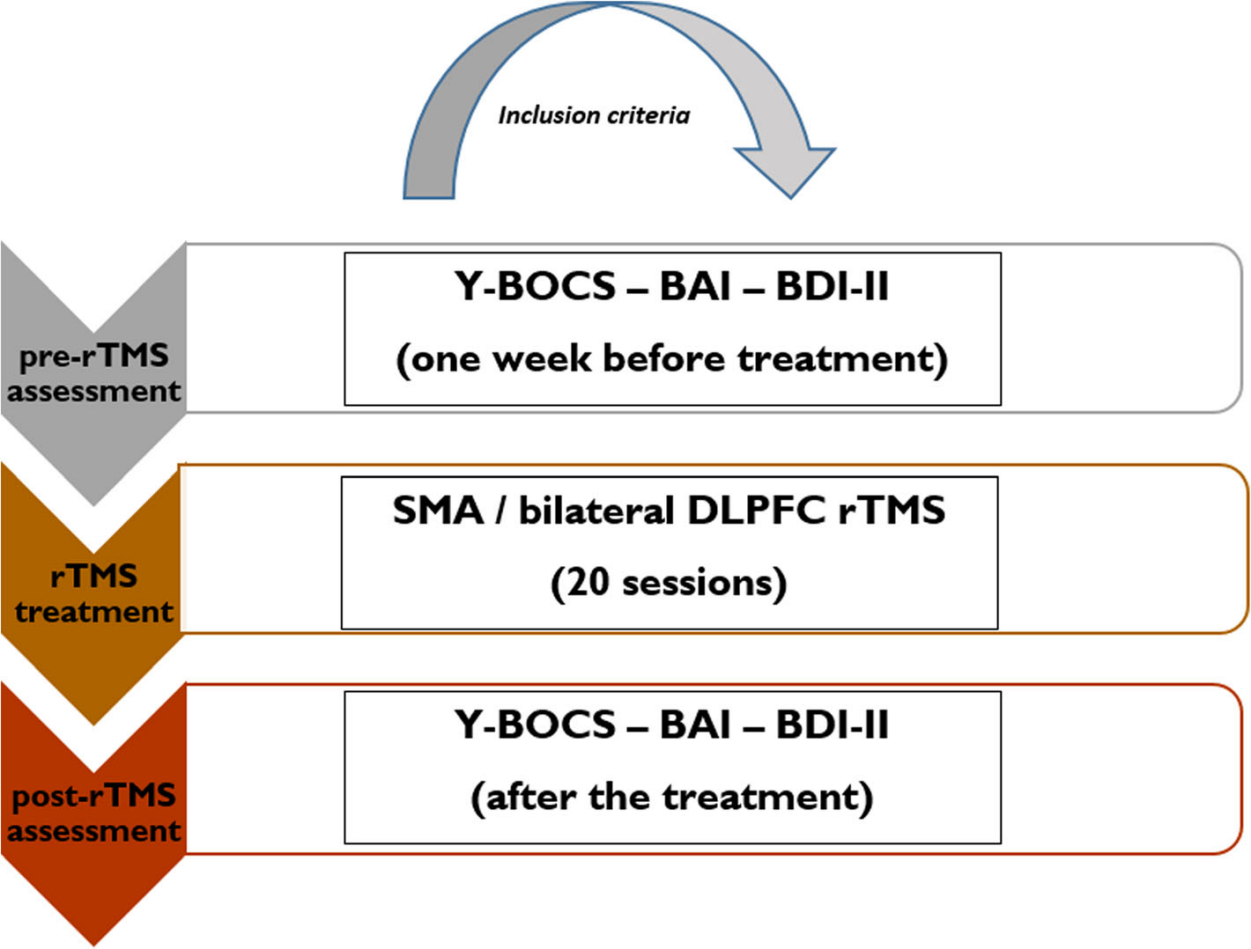
The procedure of rTMS treatment. *Note*: Y-BOCS = Yale-Brown Obsessive-Compulsive Scale; BAI = Beck Anxiety Inventory; BDI-II = Beck Depression Inventory; SMA = supplementary motor area; DLPFC = dorsolateral prefrontal cortex

All patients underwent a baseline clinical assessment with the Y-BOCS, Beck Anxiety Inventory (BAI) [45] and Beck Depression Inventory (BDI-II) [46] 1 week before rTMS treatment (pre-treatment) and after the 20th session of rTMS (post-treatment) (Fig. 1). Participants received SMA or DLPFC rTMS based on the clinical impression of a psychiatrist according to the reported symptoms. Although no significant difference was found in participants’ depressive symptoms based on the questionnaires, those who reported more depressive symptoms were allocated to DLPFC rTMS. Baseline symptom severity for inclusion was defined as a score of 16 or higher on the Y-BOCS (Mean = 22.20, *SD* = 7.01), which is the cut-off criterion for moderate OCD (8–15 mild, 16–23 moderate, 24–31 severe, 32–40 extreme). Treatment response was defined as a reduction of at least 30% in the Y-BOCS total score, based on several previous studies [33, 47] and is suggested to represent a relevant clinical improvement (i.e., improvement of Clinical Global Impression). Although 35% of symptom reduction is taken as “response” criterion in other studies [48], we chose a more liberal criterion to achieve a more balanced response distribution for the binary regression analysis. The protocol was conducted in accordance with the latest version of the Declaration of Helsinki and was approved by the Institutional Review Board and ethical committee at the local university and Atieh Clinical Neuroscience Center.

### Measures

#### Y-BOCS

The Y-BOCS is the most widely used clinician-rated interview for assessing OCD symptom severity with adequate psychometric characteristics (i.e., inter-rater reliability and predictive validity) [49]. It contains 10 items, and each item is rated from 0 (no symptoms) to 4 (extreme symptoms). The Y-BOCS is sensitive to change, and during-treatment score reductions are valid outcome indicators [49]. Therefore, results of this questionnaire are suited as clinical predictors of treatment response, as shown by previous rTMS studies [37]. The Y-BOCS items weigh obsessions and compulsions equally. Obsession items assess spent time on obsessions (item 1), interference (item 2) and distress (item 3) due to obsessive thoughts, resistance against obsessions (item 4) and degree of control over obsessive thoughts (item 5). Items 6–10 assess respective variables (i.e., spent time, interference, distress, resistance, and degree of control) for compulsions respectively.

#### BAI & BDI-II

Both BAI and BDI-II consist of 21 items, which are rated on a Likert scale ranging from 0 to 3, resulting in raw scores ranging from 0 to 63, and are indicative for the presence of anxiety or depression. The BAI is well suited to monitor anxiety treatment outcomes [50], and the obtained anxiety state is correlated with OCD symptoms [51, 52]. Similarly, the BDI-II scores are associated with OCD symptoms [52] in line with the fact that around one-third of OCD patients suffer from comorbid depression [53]. Both measures have adequate psychometric properties [54, 55].

### Statistical analysis

Data analyses were conducted using IBM, SPSS (version 24). In order to examine rTMS efficacy, mixed model analysis of variance (ANOVA) was conducted with “protocol” (DLPFC rTMS vs. SMA rTMS) as the between-subject and time (pre-intervention vs. post-intervention) as the within-subject factors. Mauchly’s test was used to evaluate sphericity of the data and in case of violation of sphericity, degrees of freedom were corrected using the Greenhouse–Geisser estimates of sphericity. The normality and homogeneity of the data were confirmed by Shapiro-Wilk and Levin tests, respectively. For identifying demographic and clinical predictors of response to rTMS treatment, participants were split into “responders” and “non-responders” and a binary logistic regression was conducted. To control for potential confounding variables, we added these variables into the model, as the model adjusts itself for potential confounders using adjusted odds-ratio [56]. The goodness of fit was evaluated by the Hosmer-Lemeshaw statistic, which also adjusts for potential covariates, and the variable selection was based on the “stepwise forward” strategy due to a large set of potential independent variables. The model was run in 2 steps in all analyses. Independent variables were age, gender, education, marital status (as demographic variables), and clinical factors were selected based on the 3-factor and 2-factor model of Y-BOCS, as well as all 10 items of the Y-BOCS that are assumed to measure different OCD symptoms. Given that the Y-BOCS items are not independent of each other, we first defined 3 and 2 factors - based on respective models derived from single items [57, 58] - as clinical predictors in separate analyses. Afterward and in a separate analysis, each individual item was treated as a potential clinical predictor of response to rTMS treatment. We ran the regression analysis separately for the demographic variables, Y-BOCS factors, and the Y-BOCS items in order not to increase the number of predictors as suggested [59]. To diagnose multicollinearity, we used the linear regression procedure and entered all covariates in the model. A significance level of *p* < 0.05 was used for all statistical comparisons.

### Data availability

The datasets used and/or analyzed for the present study are available from the corresponding author upon reasonable request.

## Results

### Participant characteristics

The mean age of responders (*N* = 30, 16 females) and nonresponders (*N* = 35, 19 females) was 32.67 (9.44), and 31.89 (10.99) years old, respectively. The groups did not differ significantly with respect to demographic variables (e.g. age, gender, education, marital status), comorbidity rate, medication use, baseline depression and applied intervention protocol (i.e., SMA vs DLPFC rTMS) (Table 1). A comparison of the same demographic variables, treatment response, and baseline OCD, anxiety and depressive scores between groups that received SMA, or vs DLPFC rTMS showed no significant differences neither (Table 2).

### Data overview

**Fig. 2:**
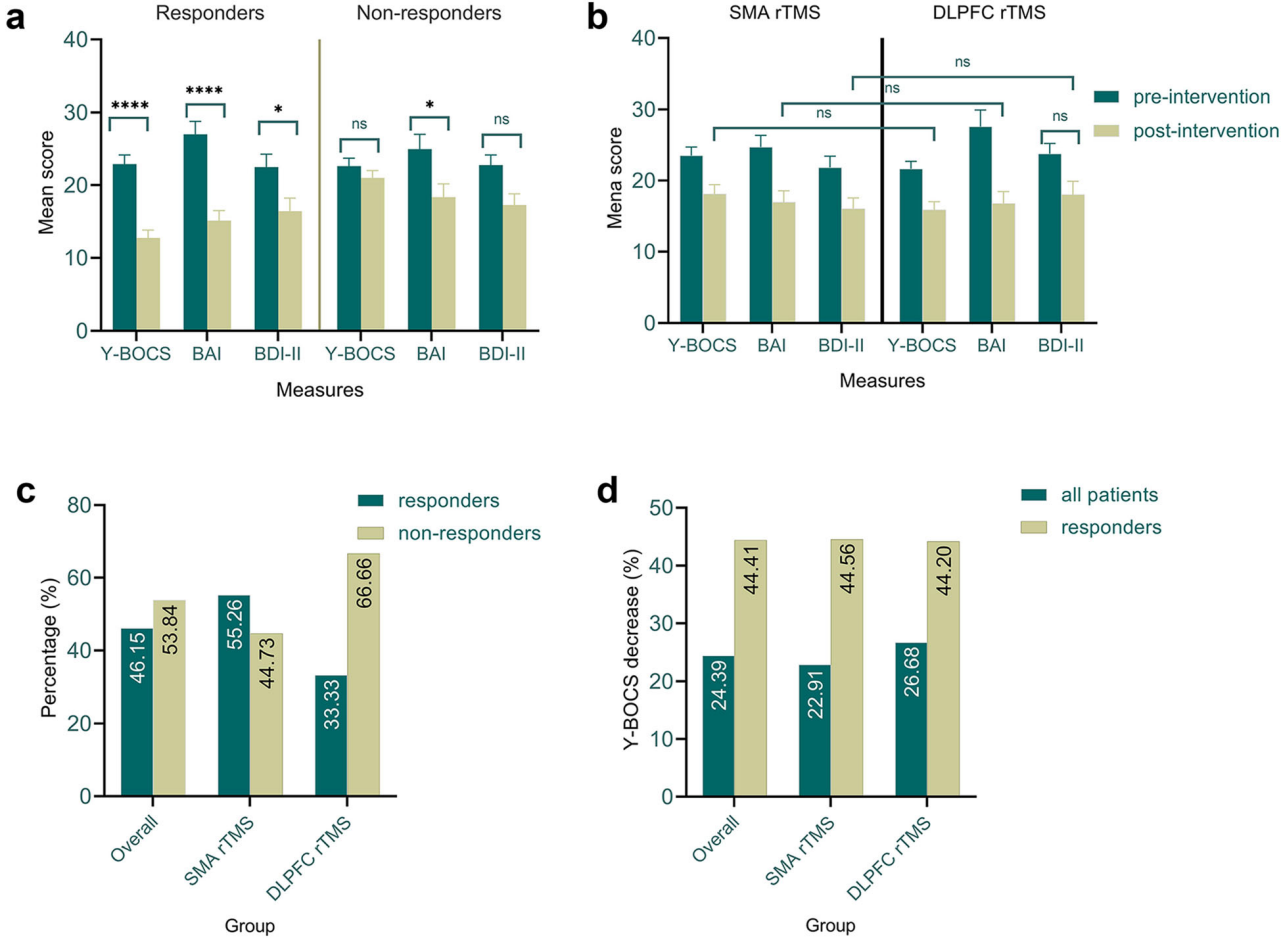
Mean score of Y-BOCS, BAI, and BDI-II before and after rTMS treatment in *responders* and *nonresponders* to rTMS (**a**). Mean score of Y-BOCS, BAI, and BDI-II before and after rTMS treatment in participants who received SMA or DLPFC rTMS (**b**). Percentage of response (**c**) and percentage of OCD symptoms decrease (**d**) in all patients and based on the intervention protocol. In graphs C and D, only post-intervention scores are compared. *Note*: Y-BOCS = Yale-Brown Obsessive-Compulsive Scale; BAI = Beck Anxiety Inventory; BDI-II = Beck Depression Inventory; * = statistically significant; ns = non-significant; All error bars represent standard error of the mean (S.E.M). Pairwise comparisons were conducted using the Bonferroni-corrected post hoc t-test

RTMS was well-tolerated by most patients. No severe adverse effects were reported except for occasional headache and dizziness, which usually disappeared spontaneously within 1–2 days. In thirty patients (46.2%), at least a 30% reduction of their Y-BOCS scores after 20 sessions of rTMS was observed. The remaining patients (53.8%) were defined as non-responders. Specifically, in patients that underwent SMA rTMS, 16 out of 38 (42.1%) responded to rTMS treatment, which is equivalent to 53.3% of all responders. From the patients received bilateral DLPFC protocol, 14 out of 27 (51.9%) responded to the rTMS treatment, which is equivalent to 46.7% of all responders (Fig. 2 C). Descriptive statistics of patients’ scores in the Y-BOCS, BAI, and BDI-II scales before and after treatment are displayed in Table 3 and Fig. 2.

**Table 3:** Means and *SD*s of OCD, anxiety and depressive scores before and after rTMS treatment based on the response (responders vs nonresponders) and protocol (SMA rTMS vs DLPFC rTMS)

Measure	Response	pre-intervention		post-intervention		Pre vs post
		***M*** (***SD***)	***t (sig)***	***M*** (***SD***)	***t (sig)***	***t (sig)***
**Y-BOCS**	responders	22.90 (6.94)	−0.32 (0.75)	12.73 (6.01)	−5.38 (**0.01**)	12.39 **(0.001)**
	non-responders	22.62 (6.50)		21.02 (5.97)		2.35 **(0.025)**
	total	22.75 (6.66)		17.20 (7.25)		7.43 **(0.001)**
	SMA rTMS	23.52 (7.37)	1.11 (0.27)	18.13 (7.95)	1.23 (0.22)	3.06 (**0.003**)
	DLPFC rTMS	21.66 (5.44)		15.88 (6.04)		3.69 (**0.001**)
**BAI**	responders	27.00 (9.72)	0.60 (0.54)	15.13 (7.51)	−0.71 (0.47)	5.84 **(0.001)**
	non-responders	24.97 (12.06)		18.37 (10.65)		2.95 (**0.005**)
	total	25.91 (11.01)		16.87 (9.41)		5.83 **(0.001)**
	SMA rTMS	24.71 (10.15)	−1.04 (0.30)	16.94 (9.95)	0.07 (0.94)	3.36 (**0.001**)
	DLPFC rTMS	27.59 (12.10)		16.77 (8.77)		3.76 (**0.001**)
**BDI-II**	responders	22.50 (9.78)	−0.20 (0.84)	16.43 (9.93)	−0.12 (0.91)	3.17 **(0.003)**
	non-responders	22.77 (8.32)		17.28 (9.16)		3.25 (**0.002**)
	total	22.64 (8.95)		16.89 (9.46)		4.57 **(0.001)**
	SMA rTMS	21.84 (9.86)	−0.85 (0.39)	16.07 (9.28)	−82 (0.41)	2.62 (**0.010**)
	DLPFC rTMS	23.77 (7.54)		18.03 (9.77)		2.42 (**0.019**)

*Y-BOCS* Yale-Brown Obsessive-Compulsive Scale, *BAI* Beck Anxiety Inventory, *BDI-II* Beck Depression Inventory, *M* Mean, *SD* Standard Deviation. Significant results are highlighted (*p* ≤ 0.05) in bold

### Effectiveness of rTMS

#### DLPFC vs SMA rTMS

No significant main effect of protocol (*F*_*(1)*_ = 1.71, *p* < 0.19, *η*_***p***_^*2*^ = 0.02) or time × protocol interaction (*F*_*(1)*_ = 0.06, *p* < 0.80, *η*_***p***_^*2*^ = 0.01) emerged, indicating no significant dfference between the protocols (i.e., SMA vs DLPFC rTMS) in reducing OCD symtoms. No significant interaction of time × protocol or main effect of protocol were found neither for the BAI scores (*F*_*(1)*_ = 0.94, *p* < 0.33, *η*_***p***_^*2*^ = 0.015; *F*_*(1)*_ = 0.43, *p* < 0.51, *η*_***p***_^*2*^ = 0.007), and depression symptoms measured by the BDI-II (*F*_*(1)*_ = 0.01, *p* < 0.99, *η*_***p***_^*2*^ = 0.001; *F*_*(1)*_ = 1.01, *p* < 0.32, *η*_***p***_^*2*^ = 0.016) (See Table 4).

**Table 4:** Results of the Mixed model ANOVAs for effects of protocol (SMA vs DLPFC rTMS) and time (pre-intervention, post-intervention) on OCD, anxiety and depressive symptoms in pharmacoresistant OCD patients

Measure	Source	df	Mean square	F	***p***-value	partial eta2
**Y-BOCS**	Time	1,63	958.15	53.51	**0.001**	0.46
	Protocol	1,63	132.82	1.70	0.197	0.01
	Time*protocol	1,63	1.15	0.06	0.803	0.05
**BAI**	Time	1,63	2727.96	34.90	**0.001**	0.36
	Protocol	1,63	58.06	0.43	0.511	0.18
	Time*protocol	1,63	73.49	0.94	0.336	0.01
**BDI-II**	Time	1,63	1044.46	19.97	**0.001**	0.24
	Protocol	1,63	119.65	1.01	0.318	0.02
	Time*protocol	1,63	0.004	0.01	0.993	0.01

*Y-BOCS* Yale-Brown Obsessive-Compulsive Scale, *BAI* Beck Anxiety Inventory, *BDI-II* Beck Depression Inventory. Significant results are highlighted (*p* ≤ 0.05) in bold

#### Overall efficacy

The results of overall ANOVA revealed a significant main effect of time on the Y-BOCS overall score in all patients (*F*_*(1)*_ = 53.51, *p* < 0.01, *η*_***p***_^*2*^ = 0.46), indicating a significant reduction of OCD symptoms after 20 sessions regardless of the stimulation protocol patiens underwent. Bonferroni-corrected post hoc paired t-tests for pre-post values showed significant differences in the Y-BOCS scores (*t* = 7.43, *p* < 0.01). With regard to anxiety and depression states, the ANOVA results similarly showed a significant main effect of time on BAI (*F*_*(1)*_ = 34.90, *p* < 0.01, *η*_***p***_^*2*^ = 0.357), and BDI-II scores (*F*_*(1)*_ = 19.97, *p* < 0.01, *η*_***p***_^*2*^ = 0.241). Again, the Bonferroni-corrected post hoc t-tests for pre-post values showed significant differences in the BAI (*t* = 5.83, *p* < 0.01) and BDI-II (*t* = 4.57, *p* < 0.01) scores.

### Predictors of rTMS treatment

Results of the multicollinearity analysis showed a variance inflation factor (VIF) of higher than 3 for only 2 dependent variables, however, a VIF around 10 is indicative for high multicollinearity [60]. The VIFs for items 1–9 were 2.60, 2.07, 3.05, 1.35, 2.98, 2.86, 3.16, 2.61, and 2.32, respectively. Since some of the individual Y-BOCS items are not completely independent from each other, we first conducted binary regression analyses on the factors extracted from the Y-BOCS items. We entered the factors from the three-factor [58] and two-factor [57] models separately as the factors from the model share similarities (Table 5). In the 3-factor model [58], regression results show that the “severity of obsessions” (Wald = 8.19) and “resistance of symptoms” factors (Wald = 5.16) were two significant predictors of rTMS treatment response in OCD. The overall model was statistically significant (χ2 = 26.64, *p* < 0.01, *df* = 2) with a Nagelkerke’s *R*^*2*^ of 0.45 and prediction success of 80% (28 of 35) and 70% (21 of 30) for non-responders and responders respectively. Regarding the 2-factor model [57], the overall model was statistically significant (χ2 = 23.71, *p* < 0.01, *df* = 1) with a Nagelkerke’s *R*^*2*^ of 0.41 and prediction success of 74.3% (26 of 35) and 76.7% (23 of 30) for non-responders and responders respectively. The Wald criterion revealed the “*disturbance*” factor (items 2,3,7,8) as the only significant clinical factor in predicting rTMS response in OCD.

**Table 5:** Binary logistic regression analysis of significant clinical predictors (Y-BOCS items and factors) of response to rTMS treatment

Predictors *Nagelkerke R*^*2*^ 0.*46(item-based analysis)*	Mean±SD item score in NR and (R)	Predicted group	***β***	Wald	df	***p***	Odd ratio (e ***β***)	95% CI	
								Lower	Upper
**Individual Y-BOCS factors**		**Non-responders**							
Interference due to obsessive thoughts (2)	2.11 ± 0.93 (1.17 ± 0.69)		1.34	9.89	1	**0.002**	3.83	1.66	8.86
Resistance against compulsions (9)	2.0 ± 1.11 (1.0 ± 0.94)		0.86	7.41	1	**0.006**	2.38	1.27	4.45
Constant			−3.31	13.06	1	0.01	0.03		
Time occupied by obsession (1)	2.40 ± 1.03 (1.53 ± 0.86)					0.10			
Distress associated with obsessions (3)	2.51 ± 0.74 (1.77 ± 1.07)					0.14			
Resistance against obsessions (4)	1.69 ± 0.90 (1.30 ± 0.79)					0.29			
Control over obsessions (5)	2.51 ± 0.88 (1.67 ± 0.84)					0.21			
Time spent on compulsions (6)	1.77 ± 0.97 (1.07 ± 0.74)					0.68			
Interference due to compulsions (7)	1.86 ± 1.06 (0.97 ± 0.92)					0.96			
Distress associated with compulsions (8)	2.03 ± 1.04 (1.03 ± 1.15)					0.81			
Control over compulsions (10)	2.17 ± 1.12 (1.23 ± 0.97)					0.67			
**2-factor Model** [57] Disturbance factor (items, 2,3,7,8)	8.51 ± 2.86 (4.93 ± 2.67)		0.51	13.41	1	**0.001**	1.67	1.27	2.20
Symptom severity factor (items 1,4,5,6,9,10)	12.54 ± 3.76 (7.80 ± 3.72)					0.09			
**3-factor model** [58] Obsession severity factor (items 1–3,5)	9.54 ± 2.74 (6.13 ± 2.90)		0.37	8.19	1	**0.004**	1.45	1.12	1.87
Resistance factor (items 4,9)	3.86 ± 1.36 (2.30 ± 1.37)		0.59	5.16	1	**0.023**	1.82	1.08	3.02
Compulsion severity factor (items 6–8,10)	7.82 ± 3.51 (4.30 ± 3.17)					0.25			

Predicted group = the group coded with value 1 in binary regression analysis; *NR* non-responders, *R* responders; Significant results are highlighted (*p* ≤ 0.05) in bold

We then conducted a separate binary logistic regression on the Y-BOCS items. The overall model was statistically significant (χ2 = 27.74, *p* < 0.01, *df* = 2), indicating that clinical predictors significantly distinguished non-responders from responders to rTMS treatment. Nagelkerke’s *R*^*2*^ of 0.46 indicates a relatively moderate relationship between predictors and rTMS response, indicating that the model explained 46% of the variance of rTMS response in OCD patients. Prediction success was 82.9% (29 of 35) and 73.3% (22 of 30) for non-responders and responders, respectively. The Wald criterion identified items 2 (interference due to obsessive thought) and 9 (resistance against compulsions) of the Y-BOCS as the two significant clinical predictors of rTMS treatment response in OCD. In other words, it was shown that an increase of 1 unit in response to items 2 and 9, would increase the risk of *not responding* to rTMS treatment 3.8 and 2.4 times (Odds ratio), respectively (Table 5). The item-based results show a pattern of response similar to the regression results of the Y-BOCS factors. We also conducted a separate binary logistic regression analysis on demographic predictors. No demographic factor, including age (*p* = 0.83), gender (*p* = 0.23), and educational level (*p* = 0.11) as well as other clinical variables such as comorbidity (*p* = 0.81) and medication use (*p* = 0.86), significantly predicted response to rTMS in OCD patients.

## Discussion

In this retrospective study, we examined rTMS therapeutic efficacy in 65 pharmaco-resistant OCD outpatients. An overall significant reduction in OCD symptoms and anxiety / depressive states were observed after 20 sessions of rTMS. 46.2% of the patients responded to rTMS treatment (based on the criterion of 30% reduction of Y-BOCS baseline scores), and a significant reduction of OCD symptoms was observed in the whole patient group, including non-responders (less than 30% symptom reduction). No significant difference was found in treatment efficacy between the intervention protocols (i.e., bilateral DLPFC vs. SMA rTMS). Regarding the predictors of rTMS response, no demographic predictor (i.e., age, gender, marital status) was identified. Both obsession and compulsion -related items/factors were related to response rate. “Obsessions severity”, “disturbance”, and “resistance” were the clinical factors that significantly predicted response to rTMS. In this line, items 2 and 9 of the Y-BOCS (i.e., “interference due to obsessive thought” and “resistance against compulsions”) were the most relevant clinical predictors of response to rTMS based on individual items regression analysis.

The response rate to rTMS in our OCD sample is in line with previous studies. The first meta-analysis in the field included 10 randomized controlled rTMS studies (with ≥25–40% reduction in Y-BOCS scores) and reported a 35% response rate in 120 OCD patients that received rTMS [20]. Other recent studies reported a response rate of 40–55% based on the 30% reduction versus Y-BOCS baseline score criterion [31–34]. In all of these studies, rTMS was applied over the DLPFC, SMA or pre-SMA except for [33], that targeted the medial PFC. Another recent meta-analysis showed that the therapeutic outcome of DLPFC vs SMA rTMS protocols is not significantly different, which was confirmed by our study results [36]. A recent rTMS study in OCD patients, which targeted the DMPFC, reported, however, a success rate of 50% with ≥50% reduction in post-treatment Y-BOCS scores specifically in those OCD patients with hyperconnectivity of fronto-striatal circuits [9].

This pattern of results suggests that response to rTMS in OCD patients depends on the pathophysiology of target region/s and the appropriate modulation of the involved regions. OCD is a heterogeneous disorder not only at the symptom, but also the pathophysiological level [4, 5, 61]. It can be speculated that nonresponders to rTMS in our study resemble OCD subtypes with specific pathophysiological features, including involved cortico-subcortical regions in deeper brain areas (e.g. OFC) that were not adequately modulated by SMA or DLPFC rTMS. The rationale for targeting SMA and DLPFC with these protocols is related to the activation pattern of these regions in OCD. The SMA and right DLPFC that have extensive connections with regions implicated in cognitive processes and motor control and response inhibition [62, 63], show hyperactivation in OCD patients. The 1 Hz stimulation applied over the SMA and right DLPFC has an inhibitory effect and is thus expected to reduce activation in these regions [43]. On the other hand, the left DLPFC is involved in cognitive control [64] and increasing its activation with NIBS has been associated with improved control over intrusive thoughts in OCD [36] and cognitive control in other disorders marked with executive dysfunctions [15, 17, 19, 65]. Increasing the left DLPFC with excitatory and right DLPFC with inhibitory stimulation is in line with the putative regions that are affected in OCD and involved in cognitive control and response inhibition/affect respectively [10]. Nevertheless, whether and how these protocols are effective in OCD patients depends on the individualized stimulation protocol taking into account the underlying pathophysiology, relevant symptoms, and comorbid diagnosis.

The length and number of rTMS sessions can also affect the response rate, with a higher number of sessions providing more symptom reduction [31, 66]. Our findings support this assumption since we observed a significant reduction of Y-BOCS scores even in non-responders with a relatively intensive intervention protocol. Here, however, results should be interpreted with caution due to the lack of sham condition which does not allow to rule out a potential placebo effect, if any, despite the baseline-control condition we have. Furthermore, similar efficacy of DLPFC vs SMA rTMS protocols should be considered in the absence of demographic and questionnaire-based group differences. Although allocation to DLPFC protocol was based on one higher report of affective states in patients, no significant difference was found between the patients in the baseline depressive scores based on the BDI-II. Future studies should apply a more strict cut-off point, using objective measures, for determining depression states and group allocation accordingly.

Our finding of the absence of relevant demographic predictors of rTMS response is in line with a recent report of a missing correlation between baseline psychometric factors, including age, and rTMS treatment outcome [9]. Age, however, seems to be a predictor of rTMS response in depression [67–70]. Regarding clinical predictors, our model showed that significant factors and items that predicted response to rTMS were related to both obsession and compulsion although the weight of obsession-related factors/items appears to be more as far as the results f the regression analyses are concerned. The *obsession severity*, *resistance* and “*disturbance*” were the factors with the highest predictive ability in rTMS response and the last two factors (resistance, disturbance factors) include items related to both obsession and compulsion. Our model based on Y-BOCS items showed a similar pattern of predictors. Specifically, we found that higher scores in “*interference* due to obsessions” (item 2), which is related to *disturbance* factor, and “*resistance* against compulsion” (item 9), related to the *resistance* factor, determined response failure to rTMS treatment. The common factor underlying both obsession and compulsion related items is the *disturbance* factor and this implicates that those OCD symptoms (including both obsession and compulsion-related ones) that result in more disturbance/interference are important in predicting response to rTMS.

The predictive value of these factors and relevant items could have clinical implications for treatment response. Patients with more “severe obsessions” have more intrusive thoughts and experience greater overall difficulties due to obsession interference [34, 71]. This is in line with recent findings showing that obsessions are usually not targeted by rTMS protocols while they are important in determining treatment response and thus should be primarily targeted in future interventions [72]. Furthermore, the relationship between intrusive thoughts and development and maintenance of OCD symptoms [73] might also explain why severe obsessive symptoms hinder response to rTMS treatment. This, however, should not implicate that compulsions are not important. Indeed, the “disturbance” factor, which is the strongest predictor among the factors, is related to distress and interference in both obsession and compulsion indicative of [57]. The “resistance” factor similarly reflects the severity of both obsessive and compulsive symptoms that are difficult to overcome [57, 58, 74]. Items related to the “resistance” factor, do not significantly change after pharmacological treatment [58, 75]. Taken together, these factors and the relevant items indicate that the severity of symptoms and the level of interference and disturbance caused by OCD symptoms (related to both obsession and compulsions) are negative predictors of rTMS response.

Since these aspects of OCD symptoms seem to be of utmost importance for response failure to rTMS treatment, it might on the one hand help to decide about therapeutic options in specific patients. On the other hand, it also suggests that patients with a lower probability for successful rTMS treatment with conventional protocols might require alternative interventions. In this line, one important implication of our findings is the need for adapting rTMS protocols to patients’ symptoms. According to our results, high levels of the interference, disturbance and resistance aspects of OCD symptoms have a negative impact on rTMS response. Patients with prominent symptoms related to these factors might benefit more from protocols that are optimized to have a strong impact on *interference* or *inhibition*. An alternative option might be to treat these patients with a treatment approach focused on improving cognitive control strategies over both obsessions and compulsions [76]. For example, treatment strategies that are focused on improving appraisal and control strategies in response to intrusive thoughts, which are associated with distress and interference [77–80], might be beneficial to OCD patients who fail to respond to rTMS due to being highly disturbed by intrusive thoughts and compulsions.

Our findings are preliminary, and this study has some limitations. The major limitation of our analysis is the retrospective study design. Nevertheless, our data has relatively high ecological validity and provides a realistic picture of rTMS application in clinical settings. Secondly, our control condition is limited to a baseline-control and lacks a sham intervention condition. Unblinded assessment by multiple raters, which could be a source of bias, and inter-rater variability, are other limitations of our work. Although the sample size was large enough for investigating the efficacy of rTMS, it may have been relatively underpowered for obtaining robust results by the regression analysis performed on individual items of the Y-BOCS. Sample size, however, was sufficiently large for factor-based regression analysis, which resulted in similar clinical predictors of rTMS response. The use of 120% of active rather than resting motor threshold could be a limiting factor in determining response rate as rTMS at AMT intensity usually delivers underdosage stimulation compared to RMT [81]. Allocation to rTMS protocols (DLPFC vs SMA) based on the prominence of depressive states determined by clinical impression and mere self-reports should be improved in future studies by applying higher cut-off points. Lastly, although we kept the medication dosage constant 8-10 weeks before the experiment and throughout the intervention (4–6 weeks) to minimize potential confounding and interference, and this factor did not predict response status, it should be controlled directly in future studies, as it might be a potential source of variability of rTMS effects.

## Conclusions

Our findings identified some important predictors of therapeutic efficacy of rTMS in OCD, which might help to develop adaptive and personalized stimulation protocols in future. Specifically, patients with severe obsession symptoms and higher dysfunctions due to intrusive thoughts, more distress and less control over compulsive behaviors might not be good candidates for receiving DLPFC and/ or SMA rTMS treatment.

## Data Availability

The datasets used and/or analyzed during the current study are available from the corresponding author upon reasonable request.

## Abbreviations

rTMS: Repetitive transcranial magnetic stimulation
OCD: Obsessive-compulsive disorder
SMA: Supplementary motor area
DMPFC: Dorsomedial prefrontal cortex
Y-BOCS: Yale-Brown Obsessive-Compulsive Scale
BAI: Beck Anxiety Inventory
BDI-II: Beck Depression Inventory
AMT: Active motor threshold
Hz: Hertz
